# 4-Amino-TEMPO loaded liposomes as sensitive EPR and OMRI probes for the detection of phospholipase A2 activity

**DOI:** 10.1038/s41598-023-40857-4

**Published:** 2023-08-22

**Authors:** Diego Alberti, Eric Thiaudiere, Elodie Parzy, Sabrina Elkhanoufi, Sahar Rakhshan, Rachele Stefania, Philippe Massot, Philippe Mellet, Silvio Aime, Simonetta Geninatti Crich

**Affiliations:** 1 Department of Molecular Biotechnology and Health Sciences, University of Torino, Via Nizza 52, 10126 Turin, Italy; 2 Univ. Bordeaux, CNRS, CRMSB, UMR 5536, 33000 Bordeaux, France; 3 Department of Science and Technological Innovation, University of Eastern Piedmont “Amedeo Avogadro”, Alessandria, Italy; 4 INSERM, Bordeaux, France; 5 IRCCS SDN SYNLAB, Via Gianturco 113, Naples, Italy

**Keywords:** Biomarkers, Nanoscience and technology

## Abstract

This work aims at developing a diagnostic method based on Electron Paramagnetic Resonance (EPR) measurements of stable nitroxide radicals released from “EPR silent” liposomes. The liposome destabilisation and consequent radical release is enzymatically triggered by the action of phospholipase A2 (PLA2) present in the biological sample of interest. PLA2 are involved in a broad range of processes, and changes in their activity may be considered as a unique valuable biomarker for early diagnoses. The minimum amount of PLA2 measured “in vitro” was 0.09 U/mL. Moreover, the liposomes were successfully used to perform Overhauser-enhanced Magnetic Resonance Imaging (OMRI) in vitro at 0.2 T. The amount of radicals released by PLA2 driven liposome destabilization was sufficient to generate a well detectable contrast enhancement in the corresponding OMRI image.

## Introduction

Phospholipase A2 (PLA2) is a large superfamily of proteins with a hydrolytic activity against phospholipids. They can selectively cleave fatty acids at the second position (sn-2) of phospholipid and are found in all mammalian tissues including plasma and serum^1,2^. PLA2 are involved in a broad range of processes, and changes in their activity may be considered as a unique valuable biomarker for early diagnoses^3–5^. Furthermore, PLA2 has been investigated as therapeutic target for several inflammatory diseases and cancer^6–8^. In fact, PLA2 expression is increased in a variety of tumours, such us prostate^9,10^, breast^11^ and pancreatic^12^ cancers, and is responsible of the release of arachidonic acid. This molecule is used in the synthesis of eicosanoids and lysophospholipids which play critical roles in the initiation and modulation of oxidative stress and inflammation^13^. Moreover, it was also observed that plasma PLA2 level increases in Covid 19 patients with a concomitant dramatic depletion of plasma phospholipids concentration. The hydrolysis of phospholipids and the related formation of lyso phospholipids operated by PLA2 enzyme activity suggested a possible role of this enzyme in the progression of the pathogenesis of Covid 19^14,15^. Therefore, a comprehensive assay system, in which the activity of each of these PLA2s could be measured sensitively and selectively, is deemed important to get more insight in to improve the understanding of their roles in a number of physiological and pathophysiological processes. Moreover, it may be useful to find new therapeutic strategies based on the use of exogenous PLA2 (e.g. from snake venom)^16^. Although many assays for PLA2 detection have already been proposed^17^, only one was recently introduced in the clinical practice in US for the prediction of patients at higher risk of coronary heart disease^18,19^. On this basis, much attention has been devoted to the development of fast, low cost and easy to use methods to perform an in vitro screening of PLA2 activity. Among the currently available methods, those based on fluorescence detection are the most sensitive ones, showing Limit of Detections (LoD) < 10 U/L^17^. They rely either on the cleavage of fluorescent molecules from properly designed systems^20,21^ and on the membrane permeabilization of lipid vesicles to remove fluorescence “quenching” effects^8,22–25^. The main disadvantages of the fluorescent based detection methods deal with the high background signal due to the autofluorescence of biological samples as well the poor penetration of the light into the opaque samples represented by cells and tissues extracts re-suspended in aqueous media. Moreover, it is well known that the limited light penetration into biological tissues is a major challenge to an efficient use of Optical Imaging in in vivo clinical diagnostic applications. In this context, we deemed of interest to look at an Electron Paramagnetic Resonance (EPR) approach, as this highly sensitive magnetic resonance technique may offer an alternative detection method for the design of PLA2 enzymatic assay that can be translated for in vivo applications using low-frequency EPR (below 1 GHz). It is worth of note to recall that EPR, when used in combination with site directed spin labelling, is commonly used to study lipid membrane dynamics as well as the structure and conformational dynamics of important membrane protein systems without size limit^26^. EPR has the advantage of being highly sensitive with limited interferences from the matrix, also in the presence of heterogeneous samples, as in the case of cell extracts and tissues. Another important characteristic of EPR probes relies on the fact that their signals may display concentration dependent “quenching” properties analogous to those ones shown by fluorescent molecules. In fact, when nitroxide radicals are loaded at high concentration in the interior of intact vesicles such as liposomes, their EPR signal becomes extremely low or undetectable^27^. On this basis, our work aimed at developing an in vitro diagnostic EPR method based on the measurements of stable 4-amino-TEMPO (TMN) radicals released by “EPR silent” liposomes thanks to PLA2 activity. EPR enzymatic assays were already reported in the literature^28–30^ but this is the first example that deals with a liposomal *on/off* EPR probe for the PLA2 activity detection. Finally, the use of radical-based probes may open new avenues for in vivo detection of the enzyme activity in a pathological tissue by means of in vivo Overhauser-enhanced Magnetic Resonance Imaging (OMRI)^31–36^. The OMRI effect consists of an “on-demand” enhancement of the solvent water signal intensity in MR images, through the electron-proton polarization transfer generated by the saturated EPR resonance of the unpaired electrons of the nitroxide radicals. The saturation of the EPR signal is obtained through the continuous wave irradiation of the EPR signal itself^37,38^. Thus, OMRI can be used to identify enzymatic activity also in vivo*,* provided that the EPR signal of the nitroxide probe changes during the catalyzed conversion to the reaction product. The potentiality of this approach was already demonstrated by the successful assessment at 0.194 T of the in vitro activities of neutrophil elastase^35,39^, pancreatic elastase (in mouse intestine)^40^ and neutrophil elastase in mouse inflamed lungs^41^ activities. In this study, preliminary results on the responsiveness of this liposomal EPR probe in OMRI experiments are also reported.

## Result and discussion

### Liposome formulation and loading of the nitroxide containing probe

**Figure 1:**
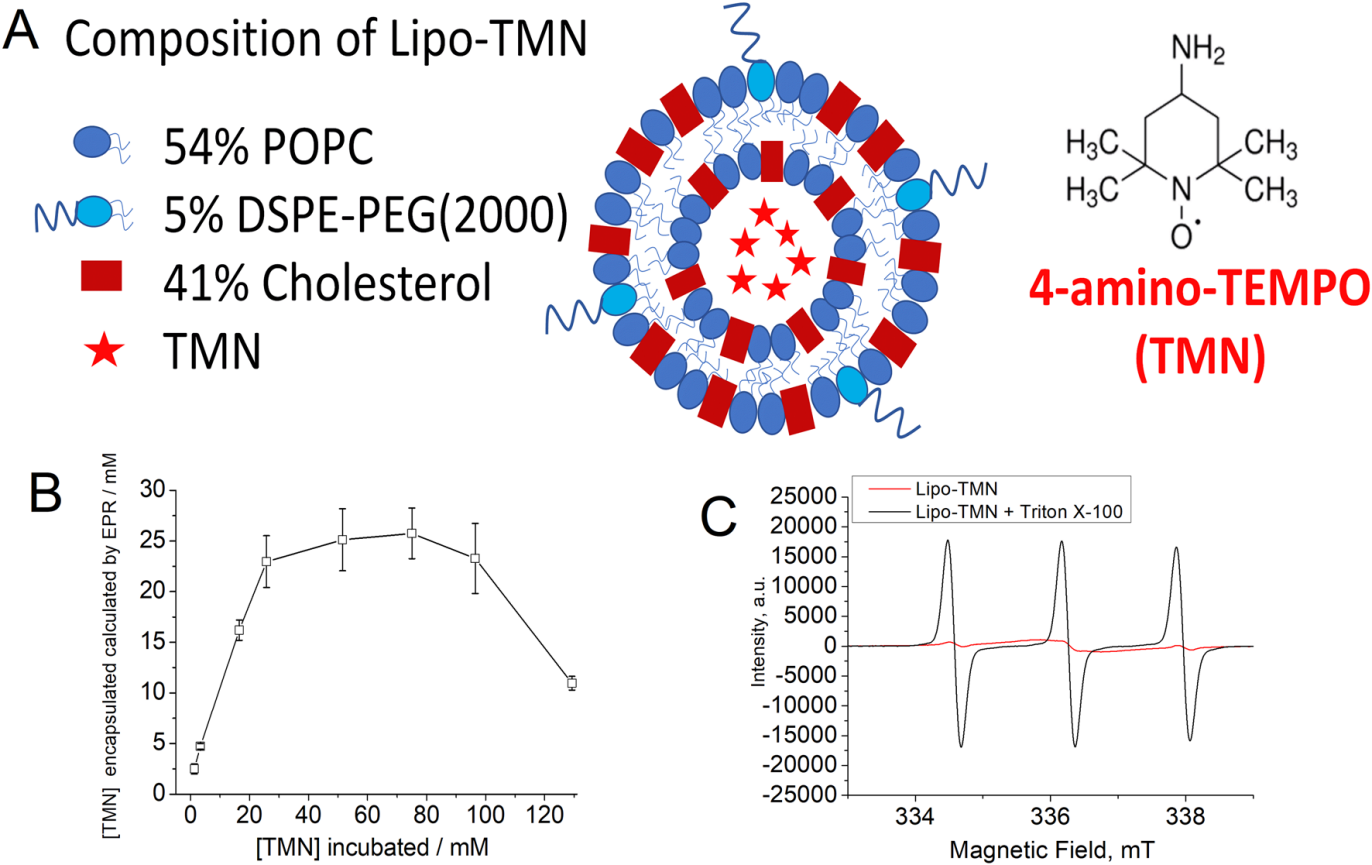
(**A**) Schematic representation of Lipo-TMN. (**B**) TMN encapsulation in Lipo-TMN calculated from the intensity of EPR signal after treatment with Triton-X100. (**C**) EPR spectra obtained on a bench-top EPR spectrometer (Spinscan X, Adani) of the intact liposome (25 mM TMN) diluted 1:20 in 0.15 M NaCl/Hepes buffer (pH 7.4) (red line) and after the addition of TRITON-X100 (black line).

TMN-loaded liposome (Lipo-TMN) was conceived with the aim of finding a system that combines a high stability, a good loading of TMN, and, at the same time, an efficient degradation in the presence of PLA2^4,5,42^. The preparation of Lipo-TMN was carried out using the thin-film hydration method followed by extrusion. POPC (2-Oleoyl-1-palmitoyl-sn-glycero-3-phosphocholine), Cholesterol and DSPE-PEG(2000) ({1,2-distearoyl-sn-glycero-3-phosphoethanolamine-N [methoxy (polyethylene glycol)-2000] ammonium salt}) were mixed at a molar ratio of 54:41:5, respectively (Fig. 1A). The addition of DSPE-PEG not only improves the stealth properties of the liposome (circulation time, stability and targeting capability), but also acts as “helper lipid” altering the structure of membrane surface allowing an increased PLA2 binding and activity^43^. This formulation is very similar to Doxil, the first FDA-approved nano-drug in which the efficient loading of doxorubicin in the liposome aqueous interior was obtained in terms of the combination of a (NH_4_)_2_SO_4_ gradient, lipid composition and temperature^44^. In Doxil, the high loading is the result of (doxorubicin)_2_SO_4_ intra-liposome crystallization, driven by a transmembrane ammonium sulphate gradient. This approach is not unique to doxorubicin, as the antioxidant nitroxide amphipathic TMN also showed intra-liposome precipitation in the presence of sulphate as a counter-ion^45,46^. Therefore, in this study, a high and stable radical payload into the liposomal cavity was reached using an ammonium sulphate gradient ([(NH_4_)_2_SO_4_]_liposome_>>[(NH_4_)_2_SO_4_]_medium_). The thin lipid film was hydrated with a solution of ammonium sulphate 250 mM. Then, the liposome was extruded and the gradient of ammonium sulphate concentration was established by dialysis. TMN was loaded into the liposome aqueous cavity upon incubation of the Lipo-TMN suspension for 1 h at 55 °C in the presence of 51.5 mM TMN followed by dialysis. The amount of remaining TMN resulted to be 25.1 ± 3.1 mM (yield = 48.7%) as assessed by EPR upon the disruption of the liposome suspension with Triton X-100 (1% v/v), as surfactant. Then, the obtained Lipo-TMN were characterized in terms of size, ζ Potential and phospholipids concentration. The average liposome hydrodynamic diameter measured by Dynamic Light Scattering (DLS) was 139.7 ± 8.5 nm (Fig. S1A) with an average polydispersity index (PDI) of 0.052. Lipo-TMN ζ Potential in 10 mM NaCl was − 12.7 ± 0.9 mV (Fig. S1B) and the amount of phospholipids recovered at the end of liposome preparation measured by Inductively coupled plasma mass spectrometry (ICP-MS) as elemental P was 54 ± 4%, i.e. 18.4 ± 2.0 mM in respect to 35.3 mM calculated from the starting materials. Thus, the precipitation of TMN inside the liposome cavity: (i) allowed the entrapment of a high amount of radicals; (ii) is responsible for an extensive “quenching” of the EPR signal that can be removed only upon the membrane destabilisation and consequent radical release. Different amounts of TMN were loaded in (NH_4_)_2_SO_4_-containing liposomes in order to optimize the preparation protocol. Figure 1B shows that the maximum loading capacity reached a plateau in the TMN concentration range 30–90 mM. The maximum loading % capacity resulted to be around 50% of the concentration of TMN present in the solution. Figure 1C shows the EPR spectrum of TMN after loading in the liposome inner cavity compared with the EPR spectrum obtained after the addition of Triton X-100 (1% v/v), a detergent able to destabilise the liposome membrane thus causing the release of the payload.

### Free TMN and liposome stability in 0.15 M NaCl/Hepes buffer and in human serum

**Figure 2:**
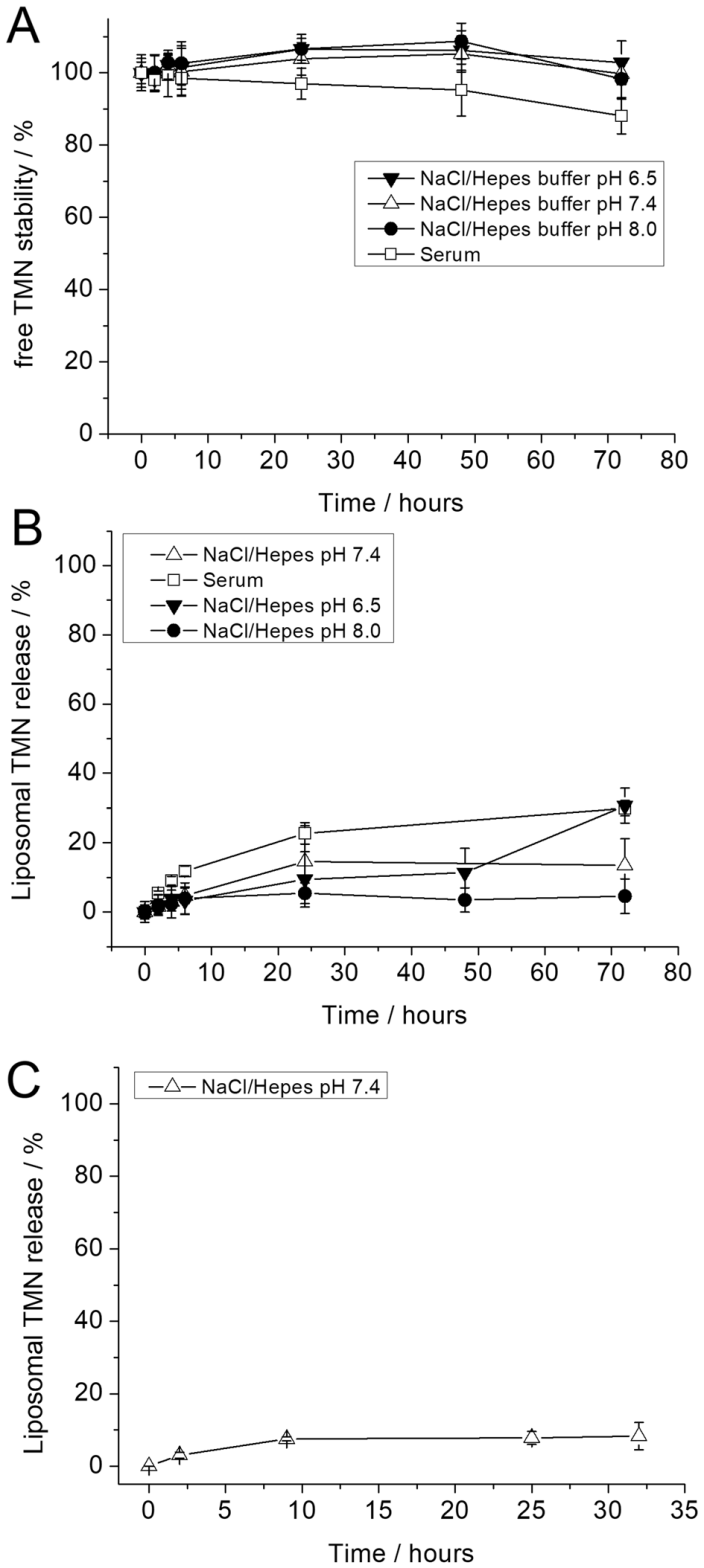
(**A**) Stability of free TMN incubated at 37 °C (400 rpm) for 72 h in 0.15 M NaCl/Hepes buffer at pH 6.5 (inverted triangle), pH 7.4 (triangle), pH 8.0 (circle) or Human Serum (square). (**B**) Percentage of liposomal TMN released by Lipo-TMN incubated at 37 °C (400 rpm) for 72 h in in 0.15 M NaCl/Hepes buffer at pH 6.5 (inverted triangle), pH 7.4 (triangle), pH 8.0 (circle) or Human Serum (square). (**C**) Percentage of liposomal TMN released by Lipo-TMN kept at 4 °C and diluted 1:20 in 0.15 M NaCl/Hepes immediately before EPR acquisition. The stability over time of free TMN was calculated as a percentage of the starting EPR intensity value (t = 0) set as 100%. The relative liposomal TMN release was calculated from the intensity of the EPR signal at each time point, compared to the EPR signal of an aliquot of the same Lipo-TMN suspension treated with Triton-X100. Since the treatment with the surfactant corresponds to the disruption of the liposome, the intensity of the resulting EPR signal was set to the value of 100% TMN released.

The stability of free TMN and of the liposomal TMN formulation were assessed at 37 °C and 4 °C by means of EPR measurements. For this purpose, free TMN and freshly prepared Lipo-TMN suspensions were incubated in 0.15 M NaCl/Hepes buffer at different pH (6.5, 7.4, 8.0) or in commercially available Human Serum (Sero, Norway) at 37 °C for 72 h. Figure 2A shows that the intrinsic stability of free TMN (not incapsulated in liposomes) was good under the applied experimental conditions, maintaining a chemical integrity of ≥ 85% over an extended period of time. Figure 2B shows that the amount of TMN released by Lipo-TMN after 72 h is ≤ 20% in NaCl/Hepes buffer (pH 7.4 and 8.0) and ~ 30% in NaCl/Hepes buffer pH 6.5 or in Human Serum. As expected the liposome stability at 4 °C is significantly higher. Figure 2C shows that the release of TMN was less than 10% after 32 days.

**Figure 3:**
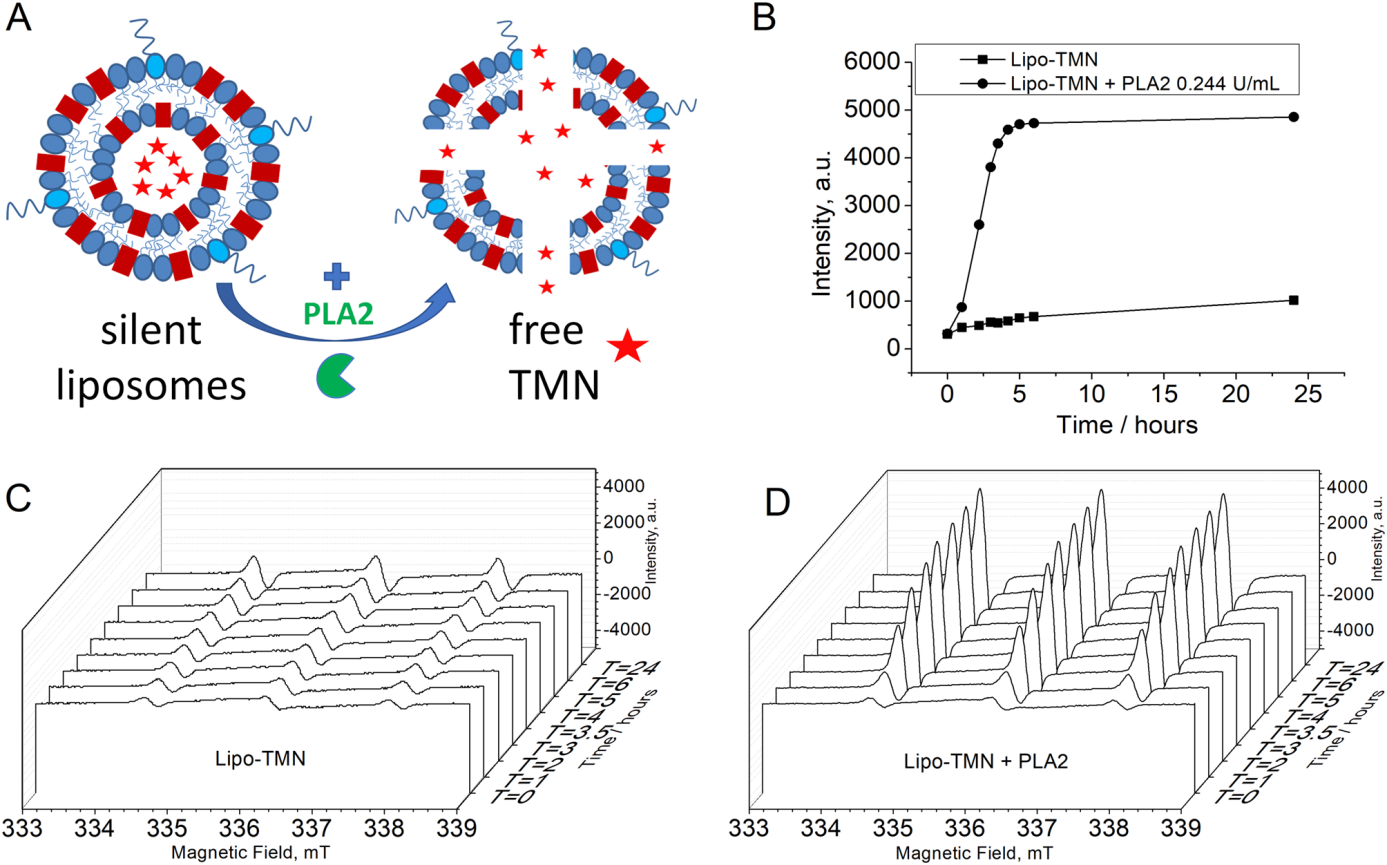
(**A**) Schematic representation of Lipo-TMN before and after the incubation with PLA2. (**B**) Plot of the EPR signal intensities upon time of the Lipo-TMN suspension (square) and in the presence of 0.244 U/mL PLA2 (27.5 nM) (circle) in 0.15 M NaCl/Hepes buffer, at 37 °C. Representative EPR spectra plots acquired from t = 0 to t = 24 h of Lipo-TMN suspensions in the absence (**C**) and in the presence of PLA2 (**D**).

The responsiveness of Lipo-TMN liposomes to enzymatic activity was assessed using PLA2 from honey bee venom (MW = 14,500 Da). The liposome suspension (25 mM in TMN) loaded with TMN diluted at 0.3 mM TMN in NaCl/Hepes buffer (pH 7.4) was incubated at 37 °C in the presence of CaCl_2_ 2 mM with or w/o PLA2 at 0.244 U/mL corresponding to the concentration of 27.5 nM PLA2. Figure 3A is a cartoon representing the Lipo-TMN before and after the action of the enzyme. As shown in Fig. 3B, the EPR signal intensity (measured on the positive derivative peak at 334.5 mT) of Lipo-TMN incubated in the presence of the enzyme increased by up to ca. 8 times in 3.5 h with respect to Lipo-TMN incubated with vehicle, reaching 4300 a.u. of ca. 4700 a.u, corresponding to plateau and 100% release of TMN. The observed behaviour was well comparable with those obtained by analysing the area under the EPR peak (Supplementary, Fig. S2). Figure 3C and D report the stack plots of the EPR spectra, from t = 0 to t = 24 h incubation time, of Lipo-TMN suspensions without and with PLA2, respectively.

### Limit of detection (LoD) of PLA2 activity

**Figure 4:**
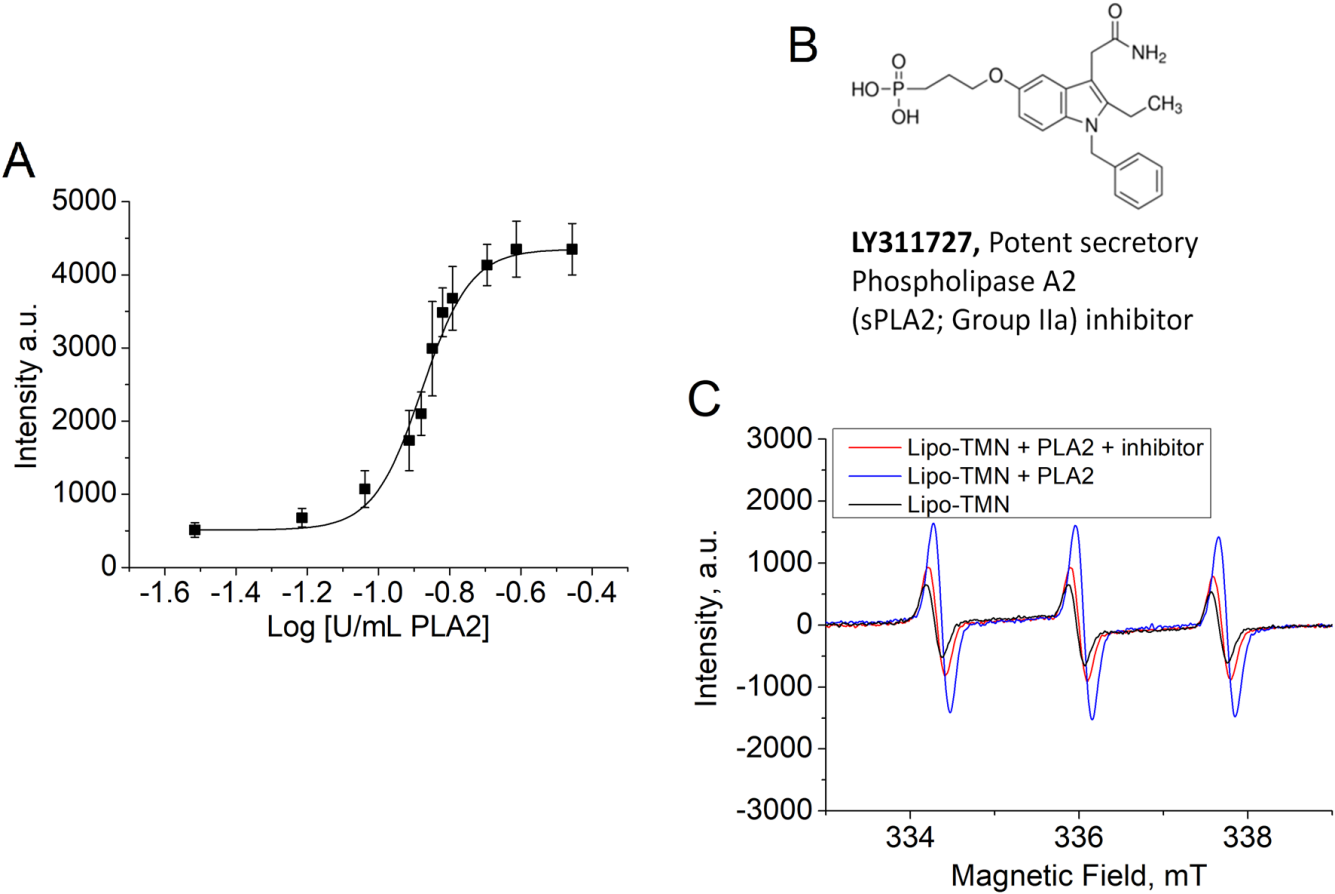
(**A**) Plot of EPR signals from Lipo-TMN incubated for 3.5 h, at 37 °C with 2mM CaCl_2_ w/o and with increasing concentration of PLA2 in 0.15M NaCl-Hepes buffer. Data are the mean ± SD of four different experiments. The continuous line corresponds to the dose response curve of the plotted data obtained by Origin 8.5 software analysis. (**B**) Chemical structure of the PLA2 inhibitor (LY311727). (**C**) EPR spectra of Lipo-TMN (black line), and Lipo-TMN incubated with PLA2, in the absence (blue line) or in the presence of the PLA2 inhibitor (red line).

To determine the sensitivity of the method, Lipo-TMN was incubated at a concentration of 0.3 mM TMN for 3.5 h, at 37 °C, and 2 mM CaCl_2_ w/o and with increasing concentration of PLA2 (0–0.35 U/mL or 0–39.4 nM) in 0.15 M NaCl/Hepes buffer (pH 7.4). 3.5 h was selected as at this time the EPR signal generated by the liposome enzymatic degradation reached the maximum value and the nonspecific release of TMN by the liposome was negligible both in buffer pH 7.4 and in Human Serum (≤ 10%). The observed EPR signal intensity exhibited a sigmoidal behaviour (Fig. 4A) showing that, only at a concentration ≥ 0.090 U/mL PLA2 (Log − 1.04), the EPR intensity is significantly different (p = 0.0177, Student t-test) from the intensity of the control (Lipo-TMN w/o enzyme), measured at t = 3.5h. The extension of the linearity range of PLA2 detection was 0.090–0.20 U/mL (Fig. S3). Using a dose–response curve analysis (Fig. 4A) a calculated EC20 (20% maximal effective concentration) of 0.10 U/mL PLA2 was then estimated. The obtained LoD demonstrated that the Lipo-TMN based assay could be sensitive enough to detect the activity of sPLA2 in several diseases^47^.

All the commercially available PLA2 assay kits used in routine practice on serum or cell samples are based on colorimetric or fluorimetric methods. For comparison, the reference linearity concentration range established in^19^ for the commercially available Diasys, Evermed, Hengxiao, and Zybio assays kits, were 184–605, 208–704, 81–328, and 273–696 U/L, respectively. All these colorimetric assays are based on the spectrophotometric detection of the 4-nitrophenol produced upon the hydrolysis of the substrate, 1-myristoyl-2-(4-nitrophenyl succinyl) phosphatidylcholine, at the sn-2 position. Other colorimetric assays^48^ are based on the hydrolysis of 1,2-dithio analog of diheptanoyl phosphatidylcholine. This substrate was used to determine PLA2 activity in several assays (e.g., bee and cobra venoms, pancreatic, etc.) with the exception of cytosolic PLA2. Upon hydrolysis of the thio ester bond at the sn-2 position by PLA2, free thiols are detected using DTNB (5,5′-dithio-bis-(2-nitrobenzoic acid) with a reference linearity range of 240–2400 U/L. The fluorimetric assay kit EnzChek^®^ appears more sensitive as it can detect bee venom PLA2 at 50 U/L. On this basis we can conclude that the herein proposed EPR method, showing a linearity range of 90–200 U/L has a sensitivity that is higher than the one shown by colorimetric tests and not too distant from the one reported for the fluorimetric assay. On this basis the method can be considered as a good alternative option to determine PLA2 concentration in serum as an early biomarker for cardiovascular and other diseases. Finally, it was measured the EPR spectra of Lipo-TMN in the presence of a specific inhibitor of PLA2 (LY311727) (Fig. 4B). Figure 4C reports on the effect of LY311727 on the activity of PLA2 (0.062 U/mL corresponding to 3.85 nM PLA2) incubated for 6 h at 37 °C in the presence of Lipo-TMN. By comparing the EPR signal intensities measured w/o and with the inhibitor incubated at a concentration of 4.2 µM, it was found that in the presence of LY311727, the activity decreased from 1700 to 920 a.u., i.e. to a value close to the intensity (650 a.u.) shown by Lipo-TMN incubated w/o PLA2.

### OMRI (experiments performed @ 0.194 T (MRI-Tech System))

**Figure 5:**
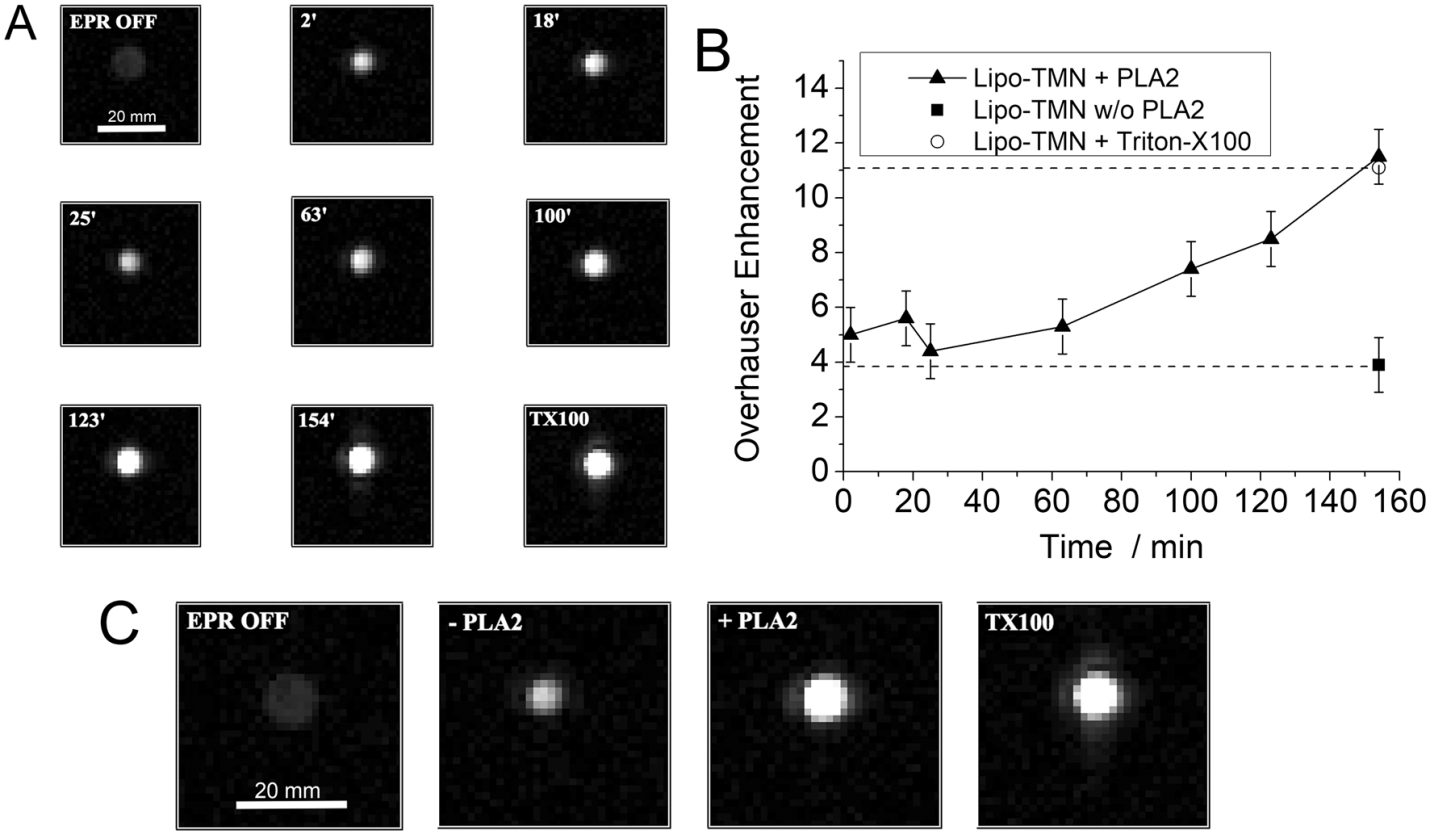
OMRI was performed with an MRI-Tech open-magnet system operating at 0.194 T. (**A**) OMR images obtained from a tube containing Lipo-TMN incubated at 37 °C in the presence of PLA2 enzyme. (**B**) Overhauser Enhancement factor calculated from OMR images upon time. (**C**) Comparison of OMR images at 150′ between Lipo-TMN with or w/o PLA2 with the one obtained with Lipo-TMN + Triton-X100. In the OMR images the white bar corresponds to 20 mm length.

On the basis of the above reported results it was deemed of interest to acquire preliminary observations with the OMRI technique. The Overhauser enhancement strictly depends on the ability to efficiently saturate the electron resonance, i.e. a condition that requires the occurrence of narrow nitroxide EPR lines. EPR spectra in Fig. 1C show that, only upon the loss of the liposome bilayer integrity, the nitroxide acquires the fast rotational regime compatible with the generation of the Overhauser effect. On the contrary, the line width of the EPR signal of intact liposome containing restrained TMN in the intraliposomal aqueous phase is definitely less suitable to generate the Overhauser effect. An in vitro OMRI test at 0.194 T was then carried out using a liposome suspension (0.6 mM in TMN) incubated at 37 °C for 150′ with or without PLA2 0.488 U/mL (55 nM PLA2). Figure 5A shows a set of images acquired with the EPR irradiation of the TMN radical as a function of time. Clearly, Fig. 5B reports the observed signal intensity increase on the release of the free radical from the liposome internal cavity. A maximum Overhauser enhancement of 11.5 (E = |ε| − 1) where ε is the dynamic nuclear polarization factor (DNPF) was obtained after 150’ incubation, only in the presence of PLA2. This value was similar to the value obtained upon liposome dissolution with Triton-X100 (E = 11.1), whereas the Overhauser Enhancement of the Lipo-TMN control solution (without PLA2) enzyme did not increase at the end of the incubation time (E = 3.9) (Fig. 5C), demonstrating the good stability of the liposome upon the time of the study.

## Conclusions

The results reported in this study demonstrate that using the commercially available radical 4-amino-TEMPO it is possible to fabricate an efficient liposome based nanoprobe for monitoring PLA2 activity. In fact, the precipitation of high amounts of TMN inside the liposome cavity involves an extensive “quenching” of the EPR signal that is removed only upon the loss of the liposome bilayer membrane integrity operated by PLA2 with the consequent radical release. The minimum amount of PLA2 measured in vitro by this method was 0.090 U/mL. The specificity of the method was assessed using the PLA2 inhibitor LY311727. Finally, the amount of radicals released by PLA2 driven liposome destabilization was sufficient to generate a well detectable contrast enhancement in the corresponding OMR images acquired at 0.194 T. At this field the EPR irradiation frequency is 5.4 GHz, i.e. a frequency corresponding to a low penetration depth which likely will limit the use of this approach to mice. Moreover, at this EPR frequency, the occurrence of tissue heating could prevent the translation of this approach to larger animals or humans. In order to overcome this problem, it was recently published by Parzy and coworkers^49,50^ a new OMRI system working at earth field, allowing a significant reduction in the value of the used EPR frequency. With this system OMRI was performed in living rats through in situ Dynamic Nuclear Polarization at 206 µT using stable and non-toxic nitroxides. In parallel, conventional images are generated at 206 µT following pre-polarization at 20 mT. At this field the EPR frequency was 70–77 MHz. The development of these prototypes working at very low field, opens new avenues for the translation of in vivo applications of enzyme responsive radicals also to larger animals and possibly to humans.

## Materials and methods

### Materials and instrumentation

4-amino-TEMPO (free radical), Phospholipase A2 from honey bee venom (Apis mellifera, product number P9279-1MG lot numbers 0000095527 and 0000131054), Cholesterol, PLA2 inhibitor LY311727, Triton-X100, NaCl, HEPES, CaCl_2_ and all the others reagents were purchased from Sigma-Aldrich. DSPE-PEG(2000) {1,2-distearoyl-sn-glycero-3-phosphoethanolamine-N [methoxy (polyethylene glycol)-2000] ammonium salt}, POPC (2-Oleoyl-1-palmitoyl-sn-glycero-3-phosphocholine) were purchased from Avanti Polar Lipids, Human serum (Seronorm Human) was purchased from Sero (Billingstad Norway), NO and ethanol absolute from VWR (Radnor, Pennsylvania, USA). The size and ζ potential were determined using dynamic light-scattering (Malvern ZS Nanosizer, UK).

### Liposome preparation and characterization

Lipo-TMN liposomes were prepared by following the thin lipid film hydration method^51^. The following liposome formulation was prepared: POPC/Cholesterol/DSPE-PEG(2000) (54/41/5 molar ratio). Lipids (80 mg) were dissolved in chloroform and a thin and dry lipid film on the bottom of a flask was obtained by the organic solvent evaporation using a rotary evaporator. Then, the lipid film was hydrated by the addition of 2 mL of ammonium sulphate solution 250 mM (Sigma-Aldrich) leading to the formation of multilamellar liposomes at a concentration of 40 mg/mL lipids. The ammonium sulphate solution is important for the 4-amino-TEMPO encapsulation in the liposome core. Then, the liposome was extruded under argon pressure (5 atm) at 55 °C at least four times using polycarbonate filters of 0.1 μm. The liposomes suspension was purified by exhaustive dialysis in a membrane (cut-off of 12,400 Da) carried out at 4 °C against using a buffer containing 0.15 M NaCl and 3.8 mM Hepes (pH = 7.4). Free radical incorporation in the internal liposome aqueous phase was performed by incubating 4-amino-TEMPO in the range 0.4–44 mg dissolved in 0.02 mL of a solution 30:70 0.9% NaCl: absolute ethanol (v/v) in a final volume of 2 mL liposome suspension (40 mg/mL lipids) for 1 h at 55 °C in a thermomixer at 400 rpm. TMN was encapsulated in the aqueous cavity of the liposome by ammonium sulphate gradient method^45^. The amount of free radical not encapsulated was removed by dialysis in NaCl/Hepes buffer (pH = 7.4). The hydrated mean diameter of liposomes was analysed at 25 °C in 0.2 µm filtered NaCl/Hepes buffer (pH 7.4). The ζ potential was analysed at 25 °C in 0.2 µm filtered 10 mM NaCl. All these samples were analysed using a dynamic light scattering (DLS) Malvern Zetasizer 3000HS (Malvern, U.K.) The polydispersity index for all the liposomes prepared in this work was smaller than 0.1. The TMN encapsulated in Lipo-TMN (40 mg/mL lipids) was measured by EPR after the addition of Triton-X100 (1% v/v) to a 20 times diluted solution of Lipo-TMN in NaCl/Hepes buffer, in order to destabilise the liposome membrane thus causing the payload release. The amount of free TMN released from Lipo-TMN was calculated from EPR spectrum by means of a calibration curve (Y = 15468X) of TMN in NaCl/Hepes buffer acquired by EPR (Intensity/a.u.) in a range 0.1–2.5 mM (Fig. S4). The amount of phospholipids recovered at the end of liposome preparation was measured by Inductively coupled plasma mass spectrometry (ICP-MS) (Element-2; Termo-Finnigan, Rodano (MI), Italy) through the determination of phosphorus (P). After adding concentrated HNO_3_ (70%) to liposome suspensions in a final volume of 0.4 mL, sample digestion was performed utilizing a high-performance Microwave Digestion System (ETHOS UP Milestone, Bergamo, Italy). The liposomes were stored at 4 °C for further experiments.

### EPR method

EPR spectra were acquired with an Adani EPR spectrometer Spinscan× (9.2–9.55 GHz) using the following parameters: center field = 336.50 mT, sweep width = 8 mT, sweep time = 30 s, modulation amplitude = 150 uT, attenuation = 20 dB, temperature = 25 °C. All the enzymatic and stability incubation were done under stirring, in Starlab Thermomixer-Mixer HC at 37 °C and 400 rpm. Solutions of liposomal or free TMN (20 µl) were placed in blaubrand intramark micropipettes (Sigma Aldrich), closed by blu-tak before EPR spectra acquisition. Then, the acquired spectra were analysed by e-Spinoza software by correcting the baseline. The EPR signal intensity used for the analysis corresponds to the TMN positive derivative peak at 334.5 mT. The area under the TMN peak at 334.5 mT (Fig. S2) was calculated by Origin 8.5 software.

### Stability studies of Lipo-TMN

To perform stability tests, free TMN 20 mM in NaCl/Hepes buffer (pH7.4) and Lipo-TMN (containing 25 mM TMN) solutions, diluted 20 times in NaCl/Hepes buffer at different pH values (pH 6.5, 7.4 and 8.0) or in Human Serum, were stirred in a thermomixer for 72 h at 37 °C, 400 rpm, (final volume 0.2 mL). At various time intervals (n = 0, 2, 4, 6, 24, 48, 72 h) EPR spectra of free TMN (1 mM) and Lipo-TMN were recorded. The stability over time of free TMN was calculated as a percentage of the starting EPR intensity value (t = 0) set as 100% stability. The percentage of the liposomal TMN released was calculated with Eq. (1):

1 $$ \% {\text{ Release }} = \, \left[ {\left( {{\text{TMN }}\left( {{\text{t}} = {\text{n}}} \right) \, {-}{\text{ TMN }}\left( {{\text{t}} = 0} \right)} \right){\text{/TNM }}\left( {{\text{tr}}} \right)} \right] \, \times { 1}00 $$

for which TMN (t = n) is the amount of free TMN calculated from recorded EPR spectrum at each time interval, TMN (t = 0) is the amount of free TMN calculated from recorded EPR spectrum at the starting point (t = 0) of the stability test and TMN (tr) is the amount of free TMN calculated from recorded EPR spectrum after treatment of Lipo-TMN with 1% (v/v) Triton-X100 that corresponds to the 100% TMN released.

Long time stability was performed by leaving Lipo-TMN (25 mM TMN) immediately after its preparation at 4 °C and at different time intervals (n = 0, 2, 9, 25, 32 day) it was diluted 20 times in NaCl/Hepes buffer and measured by EPR. The % release of TMN was calculated as explained above.

### PLA2 enzymatic assay

In order to perform enzymatic assays, the PLA2 from honey bee venom was solubilized at a concentration of 1 mg/mL in water and aliquots were prepared and stored at − 20 °C for further experiments. Kinetics study of Lipo-TMN in the presence of PLA2 was tested as follows: a Lipo-TMN solution containing TMN 25 mM was diluted in NaCl/Hepes buffer (pH 7.4) to a final TMN concentration of 0.3 mM. To this liposome suspension a 1% CaCl_2_ solution (200 mM) was added and then, it was incubated at 37 °C (400 rpm) for 24 h in NaCl/Hepes (pH 7.4) in a thermomixer in the absence or in the presence of PLA2 at 0.244 U/mL corresponding to 27.5 nM PLA2 (final volume 0.2 mL). At different time points (0, 1, 2.2, 3, 3.5, 4.2, 5, 6, 24 h) an EPR spectrum was acquired for each incubated sample till the complete release of TMN from the liposome due to the PLA2 activity.

### Limit of detection of PLA2 activity

The limit of detection of PLA2 was measured by incubating for 3.5 h Lipo-TMN at 0.3 mM TMN concentration in NaCl/Hepes buffer (pH 7.4) supplemented with 2 mM CaCl_2_ as described above, at 37 °C, w/o or with PLA2 (0.030, 0.061, 0.091, 0.122, 0.131, 0.142, 0.151, 0.161, 0.202, 0.244, 0.350 U/mL corresponding to 3.44, 6.88, 10.32, 13.75,14.96, 15.97, 17.08, 18.19, 22.73, 27.51, 39.44 nM PLA2) under stirring (400 rpm) in a thermomixer (final volume 0.2 mL). After the incubation, each sample was acquired by EPR. The EPR intensity (a.u.) of each sample was plotted against Log of PLA2 concentration. A dose–response curve was obtained and EC20 were calculated by fitting the data with an Origin 8.5 software using the Eq. (2):

2 $$ {\text{y}} = {\text{A}}1 + ({\text{A}}2 - {\text{A}}1)/(1 + 10^{{(({\text{LOG}}x0 - x)*{\text{p}})}} ) $$

where A1 and A2 are bottom and top asymptote, respectively; LOGx0 and p are center and hill slope, respectively.

### Inhibition of PLA2 activity

LY311727 (Sigma Aldrich), potent secretory Phospholipase A2 (sPLA2; Group IIa) inhibitor, was used to examine the inhibition of PLA2 activity. The PLA2 inhibition was tested by incubating for 6 h at 37 °C and 400 rpm stirring in a thermomixer, Lipo-TMN at a final concentration of 0.3 mM TMN in NaCl/Hepes buffer (pH7.4) supplemented with 2 mM CaCl_2_ w/o or with PLA2 (0.062 U/mL corresponding to 3.85 nM), the latter in the presence or in the absence of LY311727, incubated at a concentration of 4.2uM (final volume 0.2 mL). After 6 h, EPR spectrum of each sample was acquired to test the potency of the inhibitor to prevent the release of TMN thanks to PLA2 activity.

### OMRI system and acquisition

MRI was performed with a MRI-Tech open-magnet system operating at 0.194 T and capable of 20 mT/m gradient strength (MRI-Tech, Canada Inc. Edmonton, Alberta) The system was further equipped with a tunable microwave cavity operating at ca. 5.4 GHz with 25 mm aperture^32^. The microwave frequency used in this study, namely 5436 MHz corresponded to the central EPR line of the oxo-TEMPO observed with a classical X-band EPR spectrometer. The EPR cavity was also equipped with a saddle- shaped NMR coil (28 mm in diameter) operating at 8.25 MHz. Single-slice 2D gradient-echo images without (control images) and with the EPR saturation of the nitroxide line at 5436 MHz (OMR image) was acquired. Acquisition parameters: repetition time (TR): 200 ms, echo time: 20 ms; matrix size: 64 × 64; field of view: 40 mm × 40 mm; slice thickness: 5 mm; nutation angle: 30°; acquisition time: 13 s. The EPR saturation was applied throughout the acquisition.

Data Processing. Magnitude and phase images were used to calculate the DNPF defined as follows:

$$ {\text{DNPF }} =  \varepsilon \, - {\text{ 1 with }}\varepsilon  =  < {\text{Iz}} >  /{\text{ I}}_{0} $$

where < Iz > and I_0_ are the proton polarizations with and without EPR saturation, respectively. In liquids, where the electron-proton coupling factor is usually positive and lower or equal to 0.5, ε could theoretically range from 1 (no Overhauser effect) to − 110, depending on nitroxide concentration and taking into account the number of EPR lines. The negative values of ε were assessed by observing a phase shift of π in the phase image. The relationship between the Overhauser enhancement (E) and the DNPF is: Overhauser enhancement =|DNPF|− 1.

The enzyme-responsive Lipo-TMN was tested as follows: a liposome suspension (at 0.6 mM TMN concentration) was incubated at 37 °C for 154′ and stirred at 400 rpm in the presence of CaCl_2_ 2 mM with or without PLA2 0.488 U/mL, corresponding to 55 nM PLA2. At different time points (2′, 18′, 25′, 63′, 100′, 123′, 154′) the mixture of Lipo-TMN with PLA2 was transferred into a 2 cm × 0.5 cm vial and positioned at the center of the EPR cavity to perform OMRI. Moreover, the OMRI acquisition were performed on Lipo-TMN with Triton-X100 as a control reference to the 100% release of liposomal TMN and on Lipo-TMN w/o PLA2 incubated for 154′ at 37 °C.

### Supplementary Information

Supplementary Figures.

## Data Availability

The datasets used and/or analysed during the current study available from the corresponding author on reasonable request.
