# Supplementary material for: 4-Amino-TEMPO loaded liposomes as sensitive EPR and OMRI probes for the detection of phospholipase A2 activity
Source: Sci Rep. 2023 Aug 22;13:13725. doi: 10.1038/s41598-023-40857-4 (PMC10444830; doi:10.1038/s41598-023-40857-4)
Supplement: Supplementary file 1 — Supplementary Figures. [file 41598_2023_40857_MOESM1_ESM.docx]

**4-amino-TEMPO loaded liposomes as sensitive EPR and OMRI probes for the detection of phospholipase A2 activity.**

Diego Alberti^a^, Eric Thiaudiere^b^, Elodie Parzy^b^, Sabrina Elkhanoufi^a^, Sahar Rakhshan^a^, Rachele Stefania^c^, Philippe Massot^b^, Philippe Mellet^d^, Silvio Aime^e^, Simonetta Geninatti Crich^a*^

*^a^Department of Molecular Biotechnology and Health Sciences, University of Torino, Italy.*

*^b^Univ. Bordeaux, CNRS, CRMSB, UMR 5536, F-33000 Bordeaux, France.*

*^c^Department of Science and Technological Innovation University of Eastern Piedmont "Amedeo Avogadro", Alessandria, Italy.*

*^d^Univ. Bordeaux, CNRS, CRMSB, UMR 5536, F30000 Bordeaux, France;* *INSERM, Bordeaux, France.*

*^e^ Institute of Biostructures and Bioimaging (IBB), National Research Council (CNR), Torino, Italy.*

**Supplementary Materials**


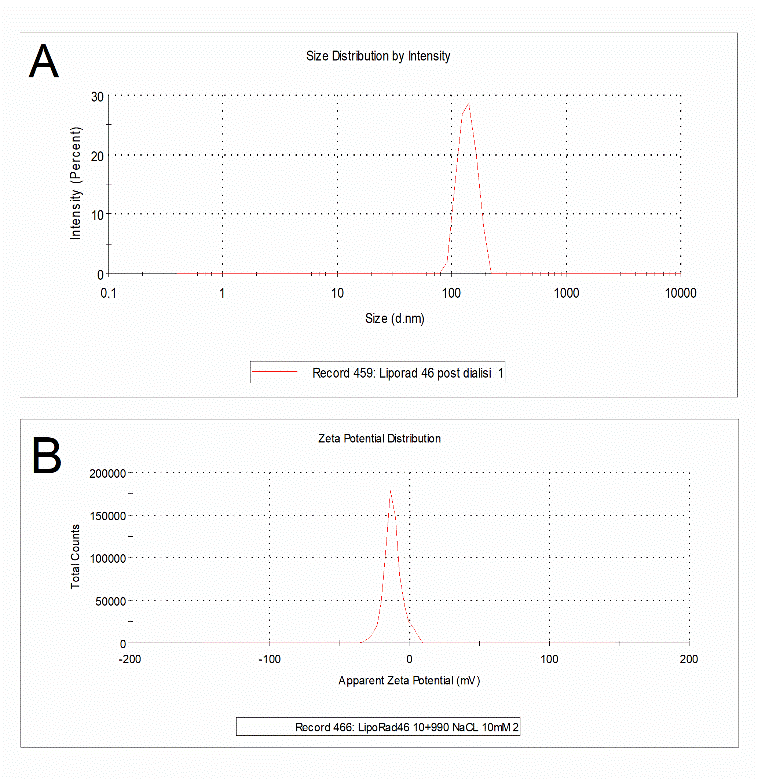


**Figure S1.** A) Representative graph of Size Distribution by Intensity of Lipo-TMN and representative graph of Zeta Potential Distribution of Lipo-TMN (B) obtained by using a dynamic light scattering (DLS) Malvern Zetasizer 3000HS (Malvern, U.K.).

**Figure S2.** Kinetics data of Lipo-TMN incubated with or w/o PLA2 for 24h at 37°C, 400 rpm stirring, acquired by EPR were analysed by considering both intensity values of the peak at 334.5 mT and the area under this peak. The area under the TMN peak at 334.5 mT was calculated by Origin 8.5 software. The two methods were compared as % enhancement with respect the value measured at t=0.





**Figure S3.** The extension of the linearity range of PLA2 detection. EPR signals from Lipo-TMN (at 0.3mM TMN) incubated for 3.5h, at 37°C and 2mM CaCl_2_ w/o and with increasing concentration of PLA2 (0.09-0.2 U/mL PLA2) in 0.15M NaCl-Hepes buffer. Data are the mean ±SD of four different experiments. The continuous line corresponds to the linear fitting of the plotted data obtained by Origin 8.5 software analysis.





**Figure S4.** Calibration curve of free TMN in NaCl/Hepes pH 7.4 in the range 0.1-2.5mM acquired by EPR. The continuous line corresponds to the linear fitting of the plotted data obtained by Origin 8.5 software analysis.
